# Antibody Persistence at 1 and 4 Years Following a Single Dose of MenAfriVac or Quadrivalent Polysaccharide Vaccine in Healthy Subjects Aged 2–29 Years

**DOI:** 10.1093/cid/civ518

**Published:** 2015-11-09

**Authors:** Aldiouma Diallo, Samba O. Sow, Olubukola T. Idoko, Siddhivinayak Hirve, Helen Findlow, Marie-Pierre Preziosi, Cheryl Elie, Prasad S. Kulkarni, Varsha Parulekar, Bou Diarra, Fadima Cheick Haidara, Fatoumata Diallo, Milagritos Tapia, Adebayo K. Akinsola, Richard A. Adegbola, Ashish Bavdekar, Sanjay Juvekar, Julie Chaumont, Lionel Martellet, Elisa Marchetti, Marc F. LaForce, Brian D. Plikaytis, Godwin C. Enwere, Yuxiao Tang, Ray Borrow, George Carlone, Simonetta Viviani

**Affiliations:** 1 Institut de Recherche pour le Développement, Niakhar, Sénégal; 2 Centre pour le Développement des Vaccins, Ministère de la Santé, Bamako, Mali; 3 Medical Research Council Laboratories, Basse, The Gambia; 4 Shirdi Sai Baba Hospital, Vadu/King Edward Memorial Hospital and Research Centre, Pune, India; 5 Vaccine Evaluation Unit, Public Health England, Manchester Royal Infirmary, United Kingdom; 6 Meningitis Vaccine Project, PATH, Ferney-Voltaire, France; 7 Meningitis Vaccine Project, Department of Immunization, Vaccines and Biologicals, World Health Organization, Geneva, Switzerland; 8 Centers for Disease Control and Prevention, Atlanta, Georgia; 9 Serum Institute of India, Ltd, Pune; 10 DiagnoSearch Life Sciences, Mumbai, India; 11 Department of Pediatrics, Center for Vaccine Development, University of Maryland School of Medicine, Baltimore; 12 GlaxoSmithKline Vaccines, Wavre, Belgium; 13 Meningitis Vaccine Project, PATH, Seattle, Washington

**Keywords:** MenA conjugate vaccine, antibody persistence, African meningitis belt, India

## Abstract

***Background.*** Mass vaccination campaigns of the population aged 1–29 years with 1 dose of group A meningococcal (MenA) conjugate vaccine (PsA-TT, MenAfriVac) in African meningitis belt countries has resulted in the near-disappearance of MenA. The vaccine was tested in clinical trials in Africa and in India and found to be safe and highly immunogenic compared with the group A component of the licensed quadrivalent polysaccharide vaccine (PsACWY). Antibody persistence in Africa and in India was investigated.

***Methods.*** A total of 900 subjects aged 2–29 years were followed up for 4 years in Senegal, Mali, and The Gambia (study A). A total of 340 subjects aged 2–10 years were followed up for 1 year in India (study B). In study A, subjects were randomized in a 2:1 ratio, and in study B a 1:1 ratio to receive either PsA-TT or PsACWY. Immunogenicity was evaluated by measuring MenA serum bactericidal antibody (SBA) with rabbit complement and by a group A–specific immunoglobulin G (IgG) enzyme-linked immunosorbent assay.

***Results.*** In both studies, substantial SBA decay was observed at 6 months postvaccination in both vaccine groups, although more marked in the PsACWY group. At 1 year and 4 years (only for study A) postvaccination, SBA titers were relatively sustained in the PsA-TT group, whereas a slight increasing trend, more pronounced among the youngest, was observed in the participants aged <18 years in the PsACWY groups. The SBA titers were significantly higher in the PsA-TT group than in the PsACWY group at any time point, and the majority of subjects in the PsA-TT group had SBA titers ≥128 and group A–specific IgG concentrations ≥2 µg/mL at any point in time in both the African and Indian study populations.

***Conclusions.*** Four years after vaccination with a single dose of PsA-TT vaccine in Africa, most subjects are considered protected from MenA disease.

***Clinical Trials Registration.*** PsA-TT-003 (ISRCTN87739946); PsA-TT-003a (ISRCTN46335400).

For almost a century, recurrent epidemics of meningitis due to group A meningococci (MenA) represent one of the major public health problems for the African meningitis belt countries [1–3]. The MenA conjugate vaccine, MenAfriVac (PsA-TT), developed within the framework of the Meningitis Vaccine Project, and manufactured by the Serum Institute of India, Ltd [3–6], was found to be safe and immunogenic in 2- to 29-year-old subjects in Mali, The Gambia, and Senegal [7], as well as in adults and children aged 2–10 years in India [8, 9]. In children aged 12–23 months, the ability of PsA-TT to prime for immunologic memory was also shown [7]. Four weeks after vaccination, PsA-TT was able to elicit serum bactericidal antibody (SBA) titers 16-fold higher than the meningococcal ACWY quadrivalent polysaccharide vaccine (PsACWY) in 12- to 23-month-old children, and almost all vaccine recipients achieved SBA titers ≥128 [7], deemed to be a discriminatory titer [10, 11]. On these grounds, and together with an excellent safety profile, the vaccine was licensed in India and prequalified by the World Health Organization (WHO). At the end of 2010, the vaccine was introduced at large scale in Burkina Faso, Mali, and Niger [12–14], the most endemic countries of the African meningitis belt, by mass vaccination campaigns targeting the 1- to 29-year-old population. Meningitis surveillance data show a dramatic reduction, or near-disappearance, of MenA in those countries where MenAfriVac has been introduced [13, 14], in concordance with the low level of MenA carriage [15, 16]. Persistence of sustained levels of antibody titers is considered to represent a key element to predict individual protection against carriage and disease. We report here antibody persistence data for 4 years after vaccination with PsA-TT in Africa and for 1 year in India.

## METHODS

The studies were designed and conducted in accordance with the Good Clinical Practice guidelines established by the International Conference on Harmonisation and with the Declaration of Helsinki and by respective country-specific regulatory authorities. Studies were approved by the competent institutional review boards as previously reported [7, 9, 17]. The International Standard randomized controlled trial number was ISRCTN87739946 for the African study (study A) and ISRCTN46335400 for the Indian study (study B).

### Study A

Subjects were recruited at the Centre pour le Développement des Vaccins, Bamako, Mali; at the Medical Research Council Laboratories, Basse, The Gambia; and at the Institut de Recherche pour le Développement, Niakhar, Senegal. The detailed study methodology has been described elsewhere [7]. In brief, subjects aged 2–29 years were recruited, stratified into 3 age groups of 2–10, 11–17, and 18–29 years, and randomized in a 2:1 ratio to receive either 1 dose of PsA-TT or a licensed PsACWY vaccine (Mencevax ACWY, GSK). Blood draws for immunogenicity evaluation were performed at baseline, 1 month (results described elsewhere) [7], 6 months, and 1 year after vaccination. Four years postvaccination, a follow-up study on immune persistence was conducted on subjects who were traced and willing to participate in the study. One blood draw was collected 4 years after vaccination.

### Study B

In study B, subjects aged 2–10 years were recruited at Shirdi Saibaba Rural Hospital, Vadu Budruk, India, and randomized in a 1:1 ratio to receive 1 dose of either PsA-TT or PsACWY. The detailed study methodology has been described elsewhere [9]. Blood draws for immunogenicity evaluation were performed at baseline, 1 month (results described elsewhere) [9], 6 months, and 1 year after vaccination.

### Immunogenicity Evaluation

The immunogenicity of PsA-TT and the group A component of PsACWY were assessed by measuring MenA SBA assay with baby rabbit complement and MenA-specific immunoglobulin G (IgG) enzyme-linked immunosorbent assay (ELISA). Measurement of SBA titers was performed at the Health Protection Agency (now Public Health England), Manchester, United Kingdom; and the ELISA was performed at the Centers for Disease Control and Prevention (CDC), Atlanta, Georgia, with the use of the standard reference serum CDC1992 [18]. The SBA reference strain was F8238, and titers were expressed as the reciprocal of the final serum dilution, resulting in a colony-count reduction of at least 50% after 60 minutes of incubation [19].

### Statistical Analysis

The difference in SBA geometric mean titers (GMTs) and group A–specific IgG geometric mean concentrations (GMCs) between the vaccine groups at 6 months and 1 year after vaccination were compared by a mixed-effects model adjusted for baseline titers or concentrations, age, sex, time, and interaction effects of interest with log_2_-transformed titers and log_10_-transformed concentrations as an outcome; the comparison was also adjusted for study site in study A. Four years postvaccination, the comparison was carried out by analysis of covariance (ANCOVA) adjusted for baseline value, sex, time, and study site. For other endpoints, the Cochran–Mantel–Haenszel test was used to compare the proportions between vaccine groups, adjusting for age in study A, and Fisher exact test was employed to compare the vaccine groups as appropriate. All immunogenicity analyses were performed in the intention-to-treat population. Missing values were treated as missing at random. Data were analyzed with SAS software, version 9.1.3.

## RESULTS

### Study A

#### Study Population

**Figure 1.**
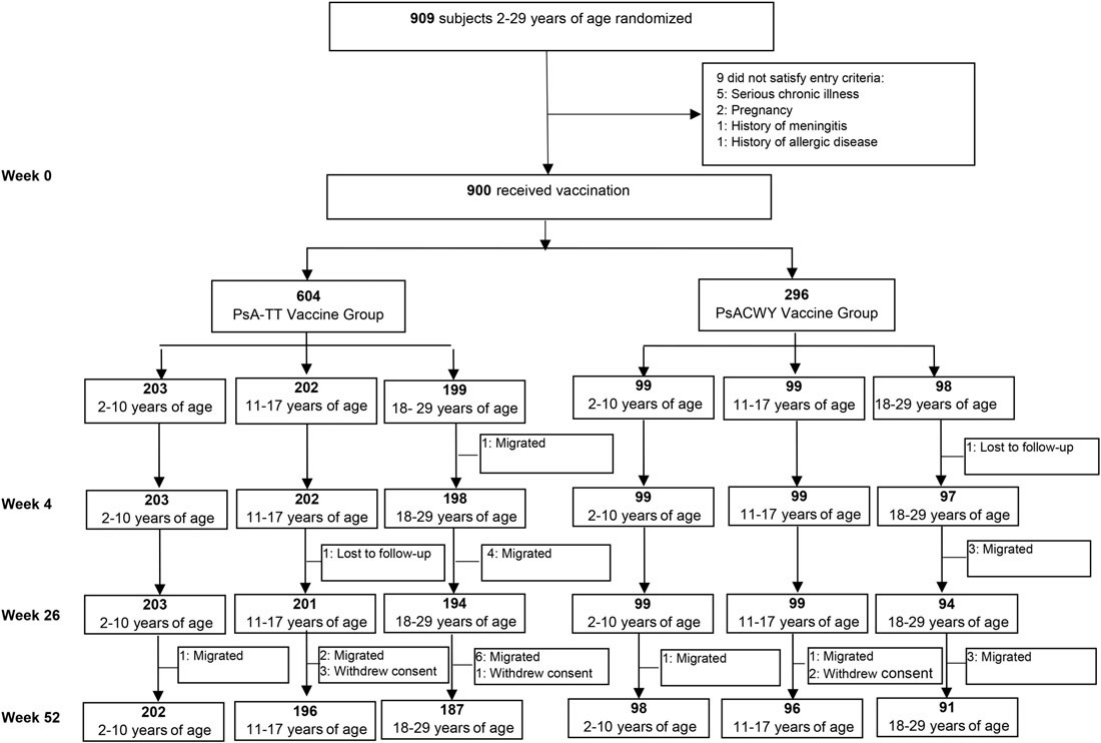
Disposition of subjects in study A conducted in healthy 2- to 29-year-olds.

Subjects' disposition during 1-year follow-up is reported in Figure 1. At 1-year postvaccination, among 900 subjects who were randomized and vaccinated at baseline, a total of 30 subjects (3.3%) were discontinued from the study. After 4 years, a total of 482 subjects (53.6% of those initially vaccinated) were available for immunogenicity—that is, with evaluable samples and who did not report, when actively questioned, additional MenA vaccination or meningococcal disease: 175 subjects aged 2–10 years (118 PsA-TT and 57 PsACWY); 149 subjects aged 11–17 years (100 PsA-TT and 49 PsACWY); and 158 subjects aged 18–29 years (110 PsA-TT and 48 PsACWY).

#### Serum Bactericidal Antibody Titers

**Figure 2.**
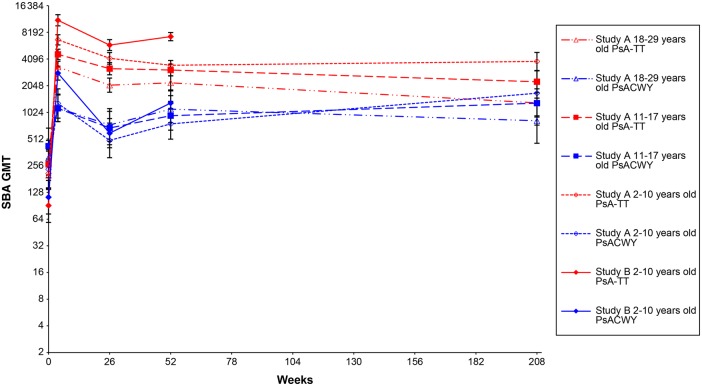
Serum bactericidal antibody (SBA) geometric mean titers (GMTs) over time for study A and study B.

SBA titers are reported in Table 1 and Figure 2.

**Table 1.** Outcomes of Meningococcal Group A Rabbit Complement Serum Bactericidal Antibody Titers for Study A Conducted in Healthy 2- to 29-Year-Olds

Age Group	Vaccine Group	Week 0 (Preimmunization)		Week 4 (28 d Postimmunization)		Week 26 (6 mo Postimmunization)		Week 52 (1 y Postimmunization)		Long-term Follow-up Study (4 y Postimmunization)	
		No.	GMT (95% CI)	No.	GMT (95% CI)	No.	GMT (95% CI)	No.	GMT (95% CI)	No.	GMT (95% CI)
rSBA GMTs											
2–10 y	PsA-TT	202	209.8 (144.4–304.7)	203	6743.2 (5900.7–7705.9)	203	4166.5 (3583.5–4844.4)	202	3485.9 (3086.4–3937.2)	118	3839.7 (3037.9–4853.2)
	PsACWY	97	233.3 (140.3–388.0)	99	1290.2 (882.4–1886.4)	98	494.2 (315.1–775.0)	98	760.8 (510.7–1133.4)	57	1685.9 (936.8–3033.9)
11–17 y	PsA-TT	202	264.9 (187.7–374.0)	202	4618.7 (4029.4–5294.2)	196	3186.5 (2718.0–3735.8)	196	3075.8 (2641.6–3581.3)	100	2272.4 (1711.4–3017.3)
	PsACWY	99	426.8 (267.7–680.4)	99	1137.4 (803.6–1609.8)	99	682.2 (442.6–1051.6)	95	938.2 (646.3–1361.7)	49	1302.4 (735.6–2305.7)
18–29 y	PsA-TT	199	199.9 (138.5–288.6)	198	3331.6 (2872.3–3864.4)	192	2077.8 (1727.7–2498.8)	186	2206.5 (1837.6–2649.4)	110	1309.3 (907.6–1888.8)
	PsACWY	97	314.9 (200.3–495.3)	95	1150.8 (805.7–1643.8)	93	737.7 (482.1–1128.9)	90	1114.5 (785.3–1581.8)	48	824.6 (458.9–1481.5)
Total	PsA-TT	603	223.3 (181.3–274.9)	603	4712.6 (4336.0–5122.0)	591	3040.8 (2758.6–3351.8)	584	2889.4 (2643.3–3158.5)	328	2281.1 (1910.5–2723.5)
	PsACWY	293	316.0 (240.4–415.3)	293	1191.4 (969.1–1464.6)	290	627.3 (488.6–805.4)	283	921.6 (743.2–1142.9)	154	1242.7 (890.7–1733.6)

		no./No.	% (95% CI)	no./No.	% (95% CI)	no./No.	% (95% CI)	no./No.	% (95% CI)	no./No.	% (95% CI)
rSBA titer ≥128											
2–10 y	PsA-TT	155/202	76.7 (70.3–82.4)	203/203	100.0 (98.2–100.0)	202/203	99.5 (97.3–100.0)	202/202	100.0 (98.2–100.0)	117/118	99.2 (95.4–100.0)
	PsACWY	77/97	79.4 (70.0–86.9)	93/99	93.9 (87.3–97.7)	84/98	85.7 (77.2–92.0)	90/98	91.8 (84.5–96.4)	53/57	93.0 (83.0–98.1)
11–17 y	PsA-TT	155/202	76.7 (70.3–82.4)	202/202	100.0 (98.2–100.0)	195/196	99.5 (97.2–100.0)	195/196	99.5 (97.2–100.0)	98/100	98.0 (93.0–99.8)
	PsACWY	85/99	85.9 (77.4–92.0)	93/99	93.9 (87.3–97.7)	89/99	89.9 (82.2–95.0)	88/95	92.6 (85.4–97.0)	46/49	93.9 (83.1–98.7)
18–29 y	PsA-TT	150/199	75.4 (68.8–81.2)	197/198	99.5 (97.2–100.0)	188/192	97.9 (94.8–99.4)	183/186	98.4 (95.4–99.7)	103/110	93.6 (87.3–97.4)
	PsACWY	81/97	83.5 (74.6–90.3)	89/95	93.7 (86.8–97.6)	85/93	91.4 (83.8–96.2)	86/90	95.6 (89.0–98.8)	44/48	91.7 (80.0–97.7)
Total	PsA-TT	460/603	76.3 (72.7–79.6)	602/603	99.8 (99.1–100.0)	585/591	99.0 (97.8–99.6)	580/584	99.3 (98.3–99.8)	318/328	97 (94.5–98.5)
	PsACWY	243/293	82.9 (78.1–87.1)	275/293	93.9 (90.5–96.3)	258/290	89.0 (84.8–92.3)	264/283	93.3 (89.7–95.9)	143/154	92.9 (87.6–96.4)

For the comparison of SBA GMTs between PsACWY and PsA-TT groups: *P* < .0001 at week 26 and week 52 after vaccination by mixed-effects model; *P* < .0001 for different age groups at week 26 and week 52. Four years after vaccination: *P* = .0002 for the comparison of SBA GMTs between PsACWY and PsA-TT groups by analysis of covariance; *P* < .0001 for age group of 2–10 years old and *P* > .05 for age groups of 11–17 years and 18–29 years. For the comparison of SBA titers ≥1:128 between PsACWY and PsA-TT groups: *P* < .0001 at week 26 and week 52 and *P* = .0350 four years after vaccination by Cochran–Mantel–Haenszel test; *P* < .05 for different age groups at week 26, week 52, and 4 years after vaccination by Fisher exact test with an exception of *P* > .05 for age groups of 11–17 years and 18–29 years 4 years after vaccination.

Abbreviations: CI, confidence interval; GMT, geometric mean titer; rSBA, rabbit complement serum bactericidal antibody.

A considerable decline in SBA GMTs was observed 6 months after vaccination in both vaccine groups, which was more marked in the PsACWY than in the PsA-TT group (approximate 62% drop in PsACWY group and 38% drop in PsA-TT group from 1 month to 6 months after vaccination). Subjects of any age group in the PSA-TT group had statistically significantly higher SBA GMTs than subjects in the PsACWY groups at any time point (*P* < .05), except at 4 years after vaccination. At 4 years postvaccination in those aged 11–17 years and 18–29 years, SBA GMTs did not significantly differ between the 2 vaccine groups. (Table 1 and Figure 2). Overall, at 1 year and 4 years after vaccination, a modest antibody decay was observed in the PsA-TT group, with a 4-year GMT of 2281.1 (95% confidence interval [CI], 1910.5–2723.5) with respect to the 1-year SBA GMT of 2889.4 (95% CI, 2643.3–3158.5) and the 6-month GMT of 3040.8 (95% CI, 2758.6–3351.8). In the PsACWY group, the SBA GMT 4 years after vaccination (1242.7 [95% CI, 890.7–1733.6]), was overall higher than the SBA GMT 1 year after vaccination (921.6 [95% CI, 743.2–1142.9]), which was also slightly higher than the GMT 6 months after vaccination (627.3 [95% CI, 488.6–805.4]). A similar trend was observed in subjects in the younger age groups of 2–10 and 11–17 years in the PsACWY group (Table 1 and Figure 2).

Six months after vaccination, the total proportion of subjects with SBA titers ≥128 was 99.0% (95% CI, 97.8%–99.6%) in the PsA-TT group and 89.0% (95% CI, 84.8%–92.3%) in the PsACWY group. In all age groups and at any time point, the proportion of subjects with SBA titer ≥128 was significantly higher in the PsA-TT group than in the PsACWY group (*P* < .05), except at 4 years after vaccination for the 2 older age groups of 11–17 years and 18–29 years. One year and 4 years after vaccination, >90% of subjects had an SBA titer ≥128 in both vaccine groups, with the significantly highest proportions observed in the PsA-TT group with 99.3% (95% CI, 98.3%–99.8%) and 97.0% (95% CI, 94.5%–98.5%), compared with 93.3% (95% CI, 89.7%–95.9%) and 92.9% (95% CI, 87.6%–96.4%) in the PsACWY vaccine group, respectively (*P* < .0001 at 1 year and *P* = .0350 at 4 years after vaccination) (Table 1).

#### GroupA–Specific IgG ELISA

**Figure 3.**
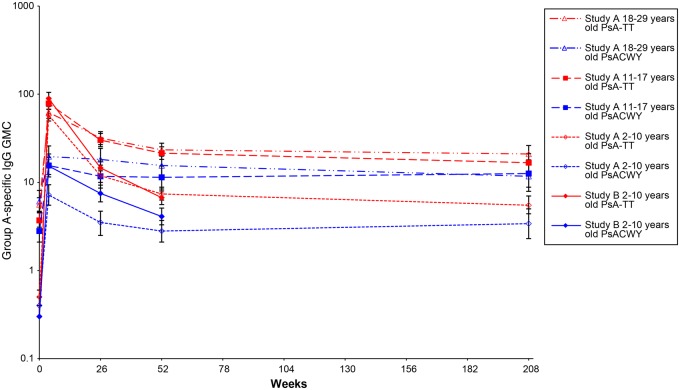
Group A–specific immunoglobulin G (IgG) geometric mean concentrations (GMCs) over time for study A and study B.

Group A–specific IgG concentrations are reported in Table 2 and Figure 3.

**Table 2.** Outcomes of Meningococcal Group A-Specific Immunoglobulin G Concentrations for Study A Conducted in Healthy 2- to 29-Year-Olds

Age Group	Vaccine Group	Week 0 (Preimmunization)		Week 4 (28 d Postimmunization)		Week 26 (6 mo Postimmunization)		Week 52 (1 y Postimmunization)		Long-term Follow-up Study (4 y Postimmunization)	
		No.	GMC (95% CI)	No.	GMC (95% CI)	No.	GMC (95% CI)	No.	GMC (95% CI)	No.	GMC (95% CI)
Group A–specific IgG GMCs											
2–10 y	PsA-TT	202	0.5 (.4–.6)	200	57.7 (49.7–67.1)	203	11.9 (10.0–14.1)	202	7.4 (6.3–8.8)	118	5.5 (4.4–7.0)
	PsACWY	97	0.4 (.3–.5)	98	7.2 (5.5–9.4)	98	3.5 (2.5–4.7)	98	2.8 (2.1–3.7)	57	3.4 (2.3–5.0)
11–17 y	PsA-TT	202	3.7 (3.1–4.5)	202	78.4 (67.5–91.1)	196	30.2 (25.4–35.9)	195	21.4 (18.2–25.3)	100	16.7 (13.1–21.2)
	PsACWY	99	2.8 (2.1–3.8)	99	15.5 (11.5–21.0)	99	11.7 (8.6–15.7)	94	11.4 (8.4–15.3)	49	12.6 (8.8–18.0)
18–29 y	PsA-TT	199	5.6 (4.7–6.7)	198	62.1 (53.1–72.7)	192	31.7 (26.8–37.6)	186	23.4 (19.8–27.8)	110	21.0 (16.9–26.2)
	PsACWY	97	6.1 (4.6–8.1)	95	19.6 (14.8–25.9)	93	18.2 (13.5–24.3)	90	15.5 (11.7–20.7)	48	11.7 (7.9–17.5)
Total	PsA-TT	603	2.1 (1.9–2.5)	600	65.6 (60.0–71.6)	591	22.3 (20.1–24.7)	583	15.3 (13.7–17.0)	328	12.1 (10.5–14.1)
	PsACWY	293	1.9 (1.5–2.3)	292	12.9 (10.9–15.3)	290	8.9 (7.4–10.8)	282	7.7 (6.4–9.3)	154	7.6 (5.9–9.6)

		no./No.	% (95% CI)	no./No.	% (95% CI)	no./No.	% (95% CI)	no./No.	% (95% CI)	no./No.	% (95% CI)
Group A–specific IgG concentration ≥2 µg/mL											
2–10 y	PsA-TT	39/202	19.3 (14.1–25.4)	200/200	100.0 (98.2–100.0)	188/203	92.6 (88.1–95.8)	174/202	86.1 (80.6–90.6)	90/118	76.3 (67.6–83.6)
	PsACWY	16/97	16.5 (9.7–25.4)	77/98	78.6 (69.1–86. 2)	65/98	66.3 (56.1–75.6)	58/98	59.2 (48.8–69.0)	37/57	64.9 (51.1–77.1)
11–17 y	PsA-TT	136/202	67.3 (60.4–73.7)	202/202	100.0 (98.2–100.0)	195/196	99.5 (97.2–100.0)	192/195	98.5 (95.6–99.7)	97/100	97.0 (91.5–99.4)
	PsACWY	56/99	56.6 (46.2–66.5)	89/99	89.9 (82.2–95.0)	87/99	87.9 (79.8–93.6)	80/94	85.1 (76.3–91.6)	45/49	91.8 (80.4–97.7)
18–29 y	PsA-TT	151/199	75.9 (69.3–81.6)	198/198	100.0 (98.2–100.0)	190/192	99.0 (96.3–99.9)	183/186	98.4 (95.4–99.7)	109/110	99.1 (95.0–100.0)
	PsACWY	77/97	79.4 (70.0–86.9)	91/95	95.8 (89.6–98.8)	87/93	93.5 (86.5–97.6)	83/90	92.2 (84.6–96.8)	42/48	87.5 (74.8–95.3)
Total	PsA-TT	326/603	54.1 (50.0–58.1)	600/600	100.0 (99.4–100.0)	573/591	97.0 (95.2–98.2)	549/583	94.2 (91.9–95.9)	296/328	90.2 (86.5–93.2)
	PsACWY	149/293	50.9 (45.0–56.7)	257/292	88.0 (83.7–91.5)	239/290	82.4 (77.5–86.6)	221/282	78.4 (73.1–83.0)	124/154	80.5 (73.4–86.5)

For the comparison of group A–specific IgG GMCs between PsACWY and PsA-TT groups: *P* < .0001 at week 26 and week 52 after vaccination by mixed-effects model; *P* < .0001 for different age groups at week 26 and week 52. Four years after vaccination, *P* < .0001 for the comparison of SBA GMTs between PsACWY and PsA-TT groups by analysis of covariance: the *P* < .05 for age groups of 2–10 years and 18–29 years and *P* > .05 for ages 11–17 years. For the comparison of group A–specific IgG concentration ≥2 µg/mL between PsACWY and PsA-TT groups: *P* < .0001 at week 26 and week 52 and *P* = .0023 four years after vaccination by Cochran–Mantel–Haenszel test; *P* < .05 for different age groups at week 26, week 52, and 4 years after vaccination by Fisher exact test with an exception of *P* > .05 for age groups of 2–10 years and 11–17 years 4 years after vaccination.

Abbreviations: CI, confidence interval; GMC, geometric mean concentration; IgG, immunoglobulin G.

Group A–specific IgG GMCs at 6 months, 1 year, and 4 years after vaccination in the PsA-TT group were 22.3 (95% CI, 20.1–24.7), 15.3 (95% CI, 13.7–17.0), and 12.1 (95% CI, 10.5–14.1) µg/mL, respectively, compared with 8.9 (95% CI, 7.4–10.8), 7.7 (95% CI, 6.4–9.3), and 7.6 (95% CI, 5.9–9.6) µg/mL in the PsACWY group, respectively. GMCs were significantly higher in the PsA-TT group than in the PsACWY group at any time point in any of the 3 age groups (*P* < .05), with an exception those aged 11–17 years 4 years after vaccination (Table 2 and Figure 3).

At 6 months, 1 year, and 4 years after vaccination, the proportions of subjects in the PsA-TT group with group A–specific IgG concentrations ≥2 µg/mL were 97.0% (95% CI, 95.2%–98.2%), 94.2% (95% CI, 91.9%–95.9%), and 90.2% (95% CI, 86.5%–93.2%), respectively, compared with 82.4% (95% CI, 77.5%–86.6%), 78.4% (95% CI, 73.1%–83.0%), and 80.5% (95% CI, 73.4%–86.5%), respectively, in the PsACWY group. The proportion of subjects with group A–specific IgG concentrations ≥2 µg/mL was significantly higher in the PsA-TT group than in the PsACWY group at any time point in any of the 3 age groups (*P* < .05), with an exception of those aged 2–10 years and 11–17 years, 4 years postvaccination (Table 2).

### Study B

#### Study Population

Demographics of study population at enrollment were reported previously [9]. In brief, a total of 340 subjects aged 2–10 years were enrolled and vaccinated, of whom 169 received PsA-TT and 171 PsACWY. Before the 6-month follow-up, 1 subject in each group was excluded from the study (1 for protocol violation, 1 withdrew consent). Therefore, 168 subjects in the PsA-TT group and 170 in the PsACWY group completed the study 1 year after vaccination.

#### Serum Bactericidal Antibody

In both vaccine groups, SBA GMTs decreased during the 6 months after vaccination, but were found to increase at 1 year of follow-up. At both time points, SBA GMTs in the PsA-TT group were significantly higher than in the PsACWY group (*P* < .0001; Figure 2 and Table 3). Six months and 1 year after vaccination, all subjects in the PsA-TT group had SBA titers ≥128, compared with 85.0% (95% CI, 78.7%–90.1%) and 93.3% (95% CI, 88.4%–96.6%), respectively, in the PsACWY group. The difference between 2 vaccine groups was significant at both time points (*P* < .0001 at 6 months and *P* = .0008 at 1 year after vaccination; Table 3).

**Table 3.** Outcomes of Meningococcal Group A Serum Bactericidal Titers for Study B Conducted in Healthy 2- to 10-Year-Olds

Vaccine Group	Week 0 (Preimmunization)		Week 4 (28 d Postimmunization)		Week 26 (6 mo Postimmunization)		Week 52 (1 y Postimmunization)	
	No.	GMT (95% CI)	No.	GMT (95% CI)	No.	GMT (95% CI)	No.	GMT (95% CI)
SBA GMTs								
PsA-TT	169	91.1 (58.7–141.3)	168	11209.1 (9708.2–12942.1)	163	5879.5 (5105.7–6770.6)	162	7329.5 (6589.2–8153.1)
PsACWY	171	112.9 (73.1–174.4)	170	2837.9 (2368.2–3400.7)	167	597.0 (409.1–871.2)	165	1306.5 (954.9–1787.6)
	no./No.	% (95% CI)	no./No.	% (95% CI)	no./No.	% (95% CI)	no./No.	% (95% CI)
SBA titer ≥128								
PsA-TT	104/169	61.5 (53.8–68.9)	168/168	100.0 (97.8–100.0)	163/163	100.0 (97.8–100.0)	162/162	100.0 (97.7–100.0)
PsACWY	111/171	64.9 (57.3–72.0)	170/170	100.0 (97.9–100.0)	142/167	85.0 (78.7–90.1)	154/165	93.3 (88.4–96.6)

For the comparison of SBA GMTs between PsACWY and PsA-TT groups: *P* < .0001 at week 26 and week 52 after vaccination by mixed-effects model. For the comparison of SBA titers ≥1:128 between PsACWY and PsA-TT groups: *P* < .0001 at week 26 and *P* = .0008 at week 52 by Fisher exact test.

Abbreviations: CI, confidence interval; GMT, geometric mean titer; SBA, serum bactericidal antibody.

#### Group A–Specific IgG

Six months after vaccination, GMCs had decreased steadily in both vaccine groups, whereas a more limited decrease was observed at 1 year. At both time points, GMCs were significantly higher in the PsA-TT group than the PsACWY group (*P* < .0001). The proportion of subjects with group A–specific IgG concentrations ≥2 µg/mL decreased over time, but was statistically significantly higher in the PsA-TT group than the PsACWY group at both time points (*P* < .0001 at 6 months and *P* = .0009 at 1 year after vaccination; Table 4 and Figure 3).

**Table 4.** Outcomes of Meningococcal Group A–Specific Immunoglobulin G Concentrations for Study B Conducted in Healthy 2- to 10-Year-Olds

Vaccine Group	Week 0 (Preimmunization)		Week 4 (28 d Postimmunization)		Week 26 (6 mo Postimmunization)		Week 52 (1 y Postimmunization)	
	No.	GMC (95% CI)	No.	GMC (95% CI)	No.	GMC (95% CI)	No.	GMC (95% CI)
Group A–specific IgG GMCs								
PsA-TT	169	0.3 (.3–0.4)	168	89.1 (75.5–105.0)	163	14.6 ( 12.3–17.4)	161	6.7 ( 5.6–8.0)
PsACWY	171	0.3 (.3–0.4)	170	15.3 (12.3–19.2)	166	7.5 ( 6.0–9.3)	165	4.1 ( 3.3–5.1)
	No.	% (95% CI)	No.	% (95% CI)	No.	% (95% CI)	No.	% (95% CI)
Group A–specific IgG concentration ≥2 µg/mL								
PsA-TT	19/169	11.2 (6.9–17.0)	168/168	100.0 (97.8–100.0)	160/163	98.2 (94.7–99.6)	137/161	85.1 (78.6–90.2)
PsACWY	20/171	11.7 (7.3–17.5)	155/170	91.2 (85.9–95.0)	142/166	85.5 (79.3–90.5)	115/165	69.7 (62.1–76.6)

For the comparison of IgG GMCs between PsACWY and PsA-TT groups: *P* < .0001 at week 26 and week 52 after vaccination by mixed-effects model. For the comparison of group A-specific IgG concentration ≥2 μg/ml between PsACWY and PsA-TT groups: *P* < .0001 at week 26 and *P* = .0009 at week 52 by Fisher exact test.

Abbreviations: CI, confidence interval; GMC, geometric mean concentration; IgG, immunoglobulin G.

## DISCUSSION

We have described 4-year antibody persistence in 2- to 29-year-old subjects in Africa and 1-year antibody persistence in 2- to 10-year-old subjects in India who had received a single dose of either PsA-TT or PsACWY. Four years after vaccination, African subjects who received PsA-TT had sustained SBA titers almost 10 times as high as the baseline values before vaccination, with the majority of subjects having SBA titers ≥128 and group A–specific IgG concentrations ≥2 µg/mL. As both these thresholds are considered potentially protective [10, 11, 20, 21], we can reasonably assume that 4 years after vaccination with the PsA-TT, the majority of subjects are still protected against MenA disease.

In both vaccine groups, SBA titers showed a substantial decline, but only during the first 6 months after vaccination. Subsequently, at 1 year and 4 years of follow-up, SBA GMTs reached a plateau, with only a modest decline compared with 6 months following PsA-TT, in those aged <18 years. The same pattern of antibody decline 6 months after vaccination was observed in the Indian subjects. However, a substantial increase in SBA titers was observed at 1 year compared with 6 months of follow-up. During the study period—4 years in The Gambia, Senegal, and Mali and 1 year in India—there was no outbreak of MenA in any of the study sites. However, the study population experienced some boosting of group A SBA, as we previously observed in a study performed in toddlers in The Gambia and Mali over approximately 1 year [7]. Exposure to cross-reacting bacteria [22–24] has been demonstrated and may explain the very high SBA titers against MenA in naive populations in the African meningitis belt [7]. In our study, exposure to cross-reacting bacteria may contribute in maintaining a sustained level of functional antibodies up to 4 years after vaccination with the MenA conjugate vaccine in Africa. The same circulating cross-reacting bacteria may well be the cause of the increase in SBA GMTs observed 1 year after vaccination in India.

SBA GMTs were higher in the PsA-TT group than in the PsACWY group, at any point in time and with any parameter considered and in both the African and Indian studies. In the African study, the age trend present in the PsA-TT group with higher SBA titers in younger subjects was conserved throughout the 4 years of follow-up. Persistence of group A IgG concentrations was also sustained over time. However, no boosting effect and no age trend were observed. Group A–specific IgG GMCs and the proportion of subjects with concentrations ≥2 µg/mL were higher in the PsA-TT group than the PsACWY group at any time point, and in both studies. A group A–specific IgG concentration ≥2 µg/mL is the only parameter validated in efficacy studies [20], and thus can be considered a putative correlate of protection against MenA. In the study in Africa, we found that 4 years after vaccination with PsA-TT, an overall 90% of subjects are still potentially protected, with the youngest age group, 2–10 years, showing the lowest level above the putative threshold of protection, with 76% of subjects having group A–specific IgG concentrations ≥2 µg/mL.

PsA-TT has already been introduced in several African meningitis belt countries, including Burkina Faso, Mali, Senegal, and The Gambia through large single-dose mass vaccination campaigns targeting the population from 1 to 29 years of age. The effect of these campaigns has been drastic, with near disappearance of cases of meningitis due to MenA as recorded by the WHO surveillance system [12–14]. Reduction of carriage of the organism is also an important parameter to demonstrate interruption of MenA transmission at the population level [15, 16].

In this context of a highly vaccinated population 4 years after vaccination with 1 dose of PsA-TT vaccine, no booster dose is required, as the majority of individuals still have the putative protective level of antibody titer [10, 11, 20, 21]. However, with time, the need for individual booster dose or revaccination of all or a portion of the population will be established by continuous monitoring of long-term antibody persistence, and by detecting any resurgence of meningitis due to group A meningococci in areas or countries where the disease was highly endemic or epidemic before introduction of PsA-TT vaccine [25].
